# Liver metabolomic profiles of sea lamprey (*Petromyzon marinus*) are influenced by sex and maturation stages

**DOI:** 10.1007/s11306-025-02266-8

**Published:** 2025-05-17

**Authors:** Sonam Tamrakar, Belinda Huerta, Yu-Wen Chung-Davidson, Weiming Li

**Affiliations:** 1 Department of Fisheries & Wildlife, Michigan State University, East Lansing, Michigan USA; 2 Chemistry Department, Southern Connecticut State University, New Haven, Connecticut USA; 3 Present Address: Proteomics and Metabolomics Facility, University of Connecticut, Storrs, Connecticut USA

**Keywords:** *Petromyzon marinus*, Untargeted metabolomics, Sex, Maturation, Bile acids, Fatty acids

## Abstract

**Introduction:**

Sea lamprey (*Petromyzon marinus)* is a unique vertebrate model to examine how liver metabolomes support different reproductive functions. Juvenile sea lamprey prey on other fish species by attaching to their body and feeding on their blood and body fluids. Once reaching adulthood, they cease feeding, migrate to spawning streams and begin their final sexual maturation. During these processes, the male livers produce large quantities of bile acid pheromone precursors to be modified and released via gills, whereas the female livers synthesize vast amounts of vitellogenin (yolk lipophosphoprotein) to be transported to the ovary.

**Objective:**

We aim to test the hypothesis that the liver metabolic pathways exhibit dramatic changes during sexual maturation of sea lampreys that support their reproductive strategies.

**Methods:**

Liver tissues from prespermiating (PSM) and spermiating (SM) males, and preovulatory (POF) and ovulatory (OF) females were homogenized, extracted and analyzed using the Thermo Q-exactive Orbitrap UPLC/MS/MS. Progenesis QI, Compound Discoverer, and Metaboanalyst were used for alignment, peak picking, deconvolution, and annotation. Data were subjected to analyses such as PCA and PLS-DA, using the SIMCA® software. The glycogen and triglyceride content in liver were also examined to determine levels of stored energy.

**Results:**

Overall, we found upregulations of amino acid and fatty acid metabolisms in mature male sea lamprey compared to the immature ones. Although the metabolic differences were comparatively subdued in the sexually immature males and females, amino acid regulation was slightly higher in females.

**Conclusion:**

We conclude that the metabolic dynamics in sea lamprey livers are consistent with their reproductive strategies.

**Supplementary Information:**

The online version contains supplementary material available at 10.1007/s11306-025-02266-8.

## Introduction

The sea lamprey is a jawless vertebrate native to the Atlantic Ocean and an invasive species in the Laurentian Great Lakes (Siefkes, 2017). They are a semelparous species, undergoing only one reproductive cycle in their lifetime. Juvenile sea lampreys parasitize for 12– 18 months on the blood and body fluids of larger fish species in the lakes or ocean. Upon reaching adulthood, they cease feeding and migrate to spawning streams for their final sexual maturation (Buchinger et al., 2015). This life cycle pattern imposes severe and unusual adaptive demands on intermediary metabolisms. On one hand, gonadal integrity must be preserved and vitellogenesis must be completed in females even after feeding has ceased. On the other hand, the energetic demands of the spawning migration must be met by catabolism of stored carbohydrates, lipids, or proteins (Sower, 1985). An upregulation in expression of genes involved in lipid metabolism, fatty acid synthesis and transport, mitochondrial function, steroid transport, and bile acid metabolism was observed in the liver of sexually mature European eels compared to immature ones (Churcher et al., 2015). However, relatively little is known about the roles of liver metabolism in lampreys when they develop through sexual maturation. During spawning migration, their livers undergo drastic changes, leading to a progressive degeneration over time. The male and female livers differ not only in size and composition but also in the rate of degeneration (Kott, 1970). Spermiating males produce large quantities of bile acid pheromone precursors in their livers, which are then modified and released via the gills to attract ovulating females (Chung-Davidson et al., 2021). The energy reserves of the females, on the other hand, is largely dedicated to producing vast amounts of vitellogenins (egg yolk lipophosphoproteins), which are transported to the ovaries. The hypotheses that adult sea lamprey adjust their metabolic pathways to support their semelparous reproductive strategy, which includes long-distance migration to spawning habitats and invest all gametes in one reproductive cycle before death, are also supported by analyses of metabolites in the plasma sampled from male and female adults (Tamrakar et al., 2022).

We predict that the liver metabolic pathways undergo significant changes during spawning migration and final sexual maturation that support their reproductive strategies. To test this hypothesis, we compared the liver metabolomes of prespermiating (PSM) and spermiating (SM) males, as well as preovulatory (POF) and ovulatory (OF) females. An LC–MS/MS-based untargeted metabolomics method was used to study the metabolic pathways influenced by the sex and maturation stage of sea lamprey in the liver extracts. We found changes in the regulation of amino acid, nucleotide, fatty acid, and bile acid metabolisms in both males and females across different stages of maturation. These findings support our hypothesis and demonstrate that liver metabolic dynamics in sea lamprey are aligned with their reproductive strategies.

## Materials and methods

### Animals

Adult sea lampreys were collected from Ocqueoc River (Presque Isle, Michigan, USA) during the migratory season in the summer of 2020 by the research staff at the US Geological Survey at Hammond Bay Biological Station, Great Lakes Science Center, Millersburg, Michigan, USA. The lampreys were grouped based on sex and maturity. Sexual maturation of pre-spawning lampreys, held in the lower Ocqueoc River, was assessed daily by visual inspection of secondary sex characteristics and gentle expression of gametes (Brant et al., 2013). Animals were then transferred to Michigan State University (East Lansing, Michigan, USA) where samples were collected immediately upon arrival. The average body length (cm) and body weight (g) of the animals were as follows (mean ± S.E.M). OF: 40.7 ± 1.4 cm, 197.4 ± 21.3 g; POF: 45.5 ± 1.2 cm, 224.3 ± 15.3 g; PSM: 44.6 ± 1.3 cm, 196.4 ± 16.8 g; SM: 43.4 ± 0.9 cm, 177.4 ± 10.9 g. Standard operating procedures for transporting, maintaining, handling, anesthetizing (with 0.2 g/L tricaine methanesulfonate, MS222), and euthanizing (with > 0.2 g/L MS222 and decapitation) sea lampreys were approved by the Institutional Committee on Animal Use and Care of Michigan State University (AUF # Li-02-17-030-99) and in compliance with standards defined by the National Institutes of Health Guide for the Care and Use of Laboratory Animals (Institute for Laboratory Animal Research, 2011). All applicable international, national, and/or institutional guidelines for the care and use of animals were followed.

### Chemicals

HPLC grade methanol, acetonitrile, chloroform, and ammonium acetate, and MS222 were purchased from Sigma-Aldrich (St. Louis, Missouri, USA). The assay kits for the analysis of glycogen and triglycerides were procured from Cayman Chemical Company (Ann Arbor, Michigan, USA).

### Sample analyses

#### Sample preparation

Sea lamprey were anesthetized with 0.2 g/L MS222, and the liver samples (99.13 ± 15.67 mg; n = 10 each for POF, PSM, OF, and SM) were dissected, snap-frozen in liquid nitrogen, and stored in − 80 °C until use. Ice-cold methanol (600 µL) was added to each sample and homogenized using a bead mill at 5 m/s for 30 s (Bead Ruptor Elite, Omni International, Inc., Kennesaw, Georgia, USA). The samples were homogenized until there were no visible chunks, with an added 30 s cycle. The liver homogenates were then incubated overnight at − 20 °C to allow complete protein precipitation, followed by high-speed centrifugation at 12,000 ×*g* at 4 °C for 15 min. The supernatant was transferred to a 15 mL tube (Corning, New York, USA). Deionized water (300 µL) and chloroform (600 µL) were added to each tube for liquid–liquid extraction. The tubes were vortexed and allowed to mix on an ice bath for 20 min at 100 rpm. This was followed by a 30 min incubation at − 20 °C, and centrifugation at 12,000 ×*g* at 4 °C for 30 min. The aqueous layer on the top was freeze-dried under vacuum and reconstituted in 50% methanol in water for LC–MS/MS analysis. The volume of solvent used for reconstitution was adjusted to obtain a final concentration of 0.5 g wet weight of liver tissue/mL. Quality control (QC) samples for male and female groups were prepared by pooling equal volumes of PSM and SM samples for male QC, and POF and OF for female QC.

#### Untargeted analyses

The untargeted metabolomics analysis was performed in a UPLC Q-Exactive Orbitrap system (Thermo Fisher Scientific Inc., Massachusetts, USA) equipped with a heated electrospray ionization source (HESI), according to a previously described method (Tamrakar et al., 2022). An Acquity UPLC BEH C18 column (100 × 2.1 mm; 1.7 µm particle) (Waters Corp., Massachusetts, USA) with 10 mM ammonium acetate in water as solvent A and acetonitrile as solvent B was used for the chromatographic separation. The mobile phase gradient was maintained as follows, 0 min: 5% B; 5 min: 38% B; 7 min: 55% B; 10 min: 70% B; 16 min: 95% B; 21 min: 100% B; 21.5– 25 min: 5% B. The flow rate was maintained at 0.3 mL/min, injection volume was 10 µL and the column temperature was set at 55 °C. Samples were analyzed using the full scan MS with data-dependent (dd)-MS2 acquisition mode in both positive and negative ionization modes. The Orbitrap parameters for the full scan acquisition were set as follows: resolution: 17,500; AGC target: 3e^6^; maximum injection time (IT): 50 ms; scan range: 80–1200 m/z. The parameters for dd-MS2 were set as follows, resolution: 17,500; AGC target: 1e^5^; IT: 50 ms; scan range: 200–2000 m/z; normalized collision energy: 10, 30, and 60. Source parameters were set as follows: sheath gas flow rate: 48 AU; spray voltage: 3.5 kV; capillary temperature: 256 °C; auxiliary gas flow rate: 11; sweep gas flow rate: 2; auxiliary gas heater temperature: 413 °C.

#### Liver glycogen and triglyceride analyses

Because glycogen and triglycerides are not readily detected in metabolomics analyses, and previous studies indicated that they play critical functions in lamprey liver during spawning migration (Bentley & Follett, 1965; Heikkala et al., 1984; Kott, 1970), we collected a portion of the liver from the same animals to measure the concentrations of liver glycogen and triglyceride, using a Glycogen Assay Kit (Cayman #700,480) and a Triglyceride Colorimetric Assay Kit (Cayman #10,010,303) according to the manufacturer’s instructions. Briefly, for the glycogen assay, liver samples were weighed (246 ± 39 mg), added with 1 mL diluted assay buffer with protease inhibitor, and homogenized using a bead mill at 5 m/s for 30 s. The homogenate was centrifuged at 800 ×*g* at 4 °C for 10 min. The supernatant was then transferred to a new tube. The supernatant (20 µL) was diluted 5 times by adding 80 µL diluted assay buffer. The diluted sample (10 µL) was then added into a well of a 96-well plate, added with 50 µL hydrolysis enzyme solution and incubated at 37 °C for 30 min. 150 µL Developer solution was then added and incubated at 37 °C for 15 min. The fluorescence (excitation: 530–540 nm; emission: 585–595 nm) was measured using a GloMax®-Multi + Detection System with Instinct® Software (Promega Corp., Madison, Wisconsin, USA), and the glycogen concentration was calculated based on a standard curve (0–40 µg/mL) assayed simultaneously.

For the triglyceride assay, liver samples were weighed (265 ± 42 mg), added with 1 mL NP40 reagent and protease inhibitor, and homogenized using a bead mill at 5 m/s for 30 s. The homogenates were centrifuged at 10,000 ×*g* at 4 °C for 10 min. The supernatant (500 µL) was transferred to a new tube and added with 500 µL Dilution Assay Buffer. The sample mixture (10 µL) was added into a well of a 96-well plate, added with 150 µL diluted enzyme mixture and incubated at room temperature for 15 min. The absorbance (530–550 nm) was measured using a GloMax®-Multi + Detection System with Instinct® Software, and the triglyceride concentration was calculated based on a standard curve (0– 200 mg/dL) assayed simultaneously.

### Data analysis

#### Untargeted metabolomics analyses

The raw data files obtained from the untargeted LC–MS/MS analysis were processed using Progenesis QI (Waters Corp.), Compound Discoverer 3.1 (Thermo Fisher Scientific Inc.), and Metaboanalyst (http://www.metaboanalyst.ca/) softwares. Raw files were converted to.mzml files using ProteoWizard (MS Convert; Chambers et al., 2012). The parameters for peak picking, retention time alignment, and deconvolution were similar to a previously described method (Tamrakar et al., 2022). Briefly, the spectra were selected from the raw data, with a filter of abundance > 10,000; and then aligned with a retention time (RT) tolerance of 0.2 min and mass error of 5 ppm. The discriminating features were selected based on the ANOVA p-value (< 0.01) and fold change (FC > 1.2). For metabolite annotation, only those features that had MS2 information were included. An added selection criteria of at least 3 database matches out of 4 (mzCloud, Metabolika, ChemSpider and predicted composition) in Compound Discoverer 3.1; and a score > 45 (out of 60) and a fragmentation score > 50 (out of 100) in Progenesis QI was applied to increase the confidence in annotations. Apart from the metabolite annotation from Progenesis QI and Compound Discoverer 3.1, the annotations of significant metabolites were based on the fragment matching with available databases, especially Human Metabolome Database (HMDB) (https://hmdb.ca/) and KEGG (https://www.genome.jp/kegg) to maximize the number of metabolite identification. Principal component analyses (PCA) and partial least squares discriminant analyses (PLS-DA) were performed by exporting the data to SIMCA 17 software (Sartorius Stedim North America Inc., NY, USA).

#### Statistical analyses

An unsupervised dimensionality-reduction tool, principal component analysis (PCA), was used to identify metabolomics data patterns and to check the trends and outliers, using SIMCA 17 software (Smilowitz et al., 2013). Data were log transformed, scaled by Pareto scaling, and subjected to autofit setting. Moreover, partial least squares discriminant analysis (PLS-DA), a supervised machine learning dimensionality-reduction tool, was also used as it is known to be well suited for metabolomics data with large number of features, noise and missing data, and fewer samples than features (Ruiz-Perez et al., 2020). The PCA and PLS-DA models were evaluated in terms of their goodness of fit (R2Xcum, R2Ycum) and goodness of prediction (Q2cum). R2Xcum is the cumulative modeled variation in X, R2Ycum is the cumulative variation in X correlated to Y, and Q2cum estimates the cumulative predictive ability of the model. ANOVA analyses were used to compare liver glycogen and triglyceride concentrations among POF, OF, PSM and SM. Bonferroni/Dunn post hoc tests were performed if ANOVA test showed significant difference among groups (p < 0.05).

#### Pathway analyses

Based on the compounds annotated, the potential impacted pathways were assessed by pathway analyses using MetaboAnalyst 4.0 (http://www.metaboanalyst.ca/), the Human Metabolome Database (HMDB, https://hmdb.ca/) (Chong et al., 2019), and Interactive Pathways Explorer (iPath) v3 (https://pathways.embl.de/). For a clearer representation of the impacted pathways, a simplified metabolic map was created based on the Kegg metabolic pathway maps by the first author, to highlight the metabolites along with their fold changes.

## Results

### Untargeted metabolomics

**Fig. 1:**
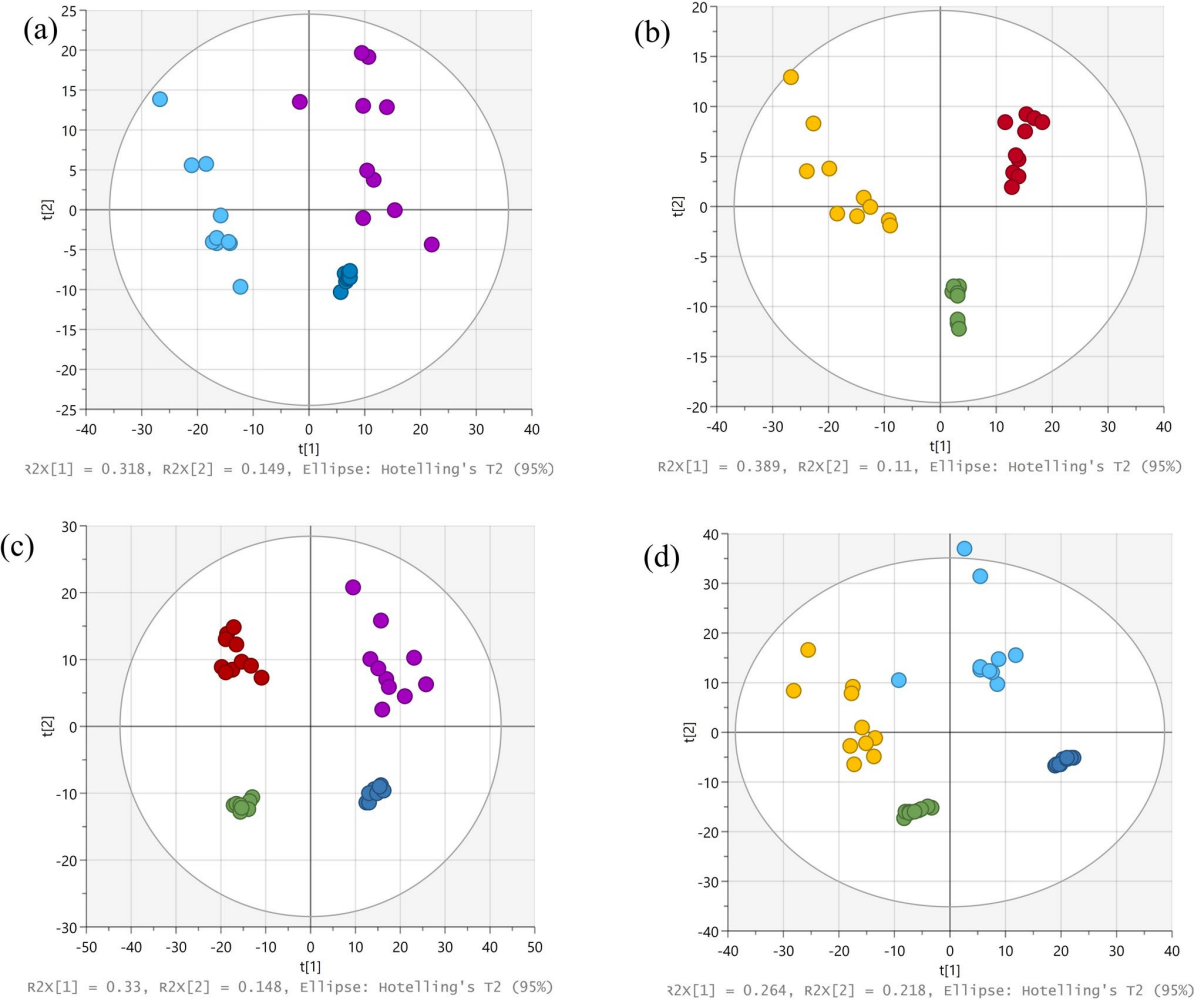
PCA score plots of the metabolic fingerprints of sea lamprey liver extracts analyzed in negative ionization mode: **a** prespermiating males (PSM) vs. spermiating males (SM); **b** preovulatory females (POF) vs. ovulatory females (OF); **c** OF vs SM; and **d** POF vs PSM. The colors of the circles represent the different sample groups: Light blue, PSM; purple, SM; yellow, POF; red, OF; green, female QC; and dark blue, male QC (Color figure online)

The score plots from the unsupervised PCA analysis of all 4 comparison groups, PSM vs. SM, POF vs. OF, SM vs. OF, and PSM vs. POF in the ESI negative mode are shown in Fig. 1. The groups showed significant separation as indicated by the goodness of fit (R2X cum) for the models. The R2X cum values for the male and female sexual maturation groups were 0.691 (PSM vs SM) and 0.638 (POF vs. OF), respectively; and that for the sex difference groups were 0.759 (PSM vs. POF) and 0.678 (SM vs. OF), respectively. The Q2 cum values ranged between 0.395 and 0.509 (Table S5).

A supervised PLS-DA analysis revealed that the extracted features were discriminants between groups. The score plots of the groups in the ESI negative ionization mode are provided as supplementary information in Fig. S1. The groups showed significant separation as indicated by the goodness of fit (R2X cum) for the models. The R2X cum values for the male and female sexual maturation groups were 0.614 (PSM vs. SM) and 0.605 (POF vs. OF), respectively; and that for the sex difference groups were 0.638 (PSM vs. POF) and 0.957 (SM vs. OF), respectively. The Q2 cum values ranged between 0.850 and 0.968 (Table S5). The goodness of fit for all groups were confirmed by R2X > 0.6, R2Y > 0.9, and Q2 > 0.8 (Table S5).

#### Prespermiating males versus spermiating males

**Fig. 2:**
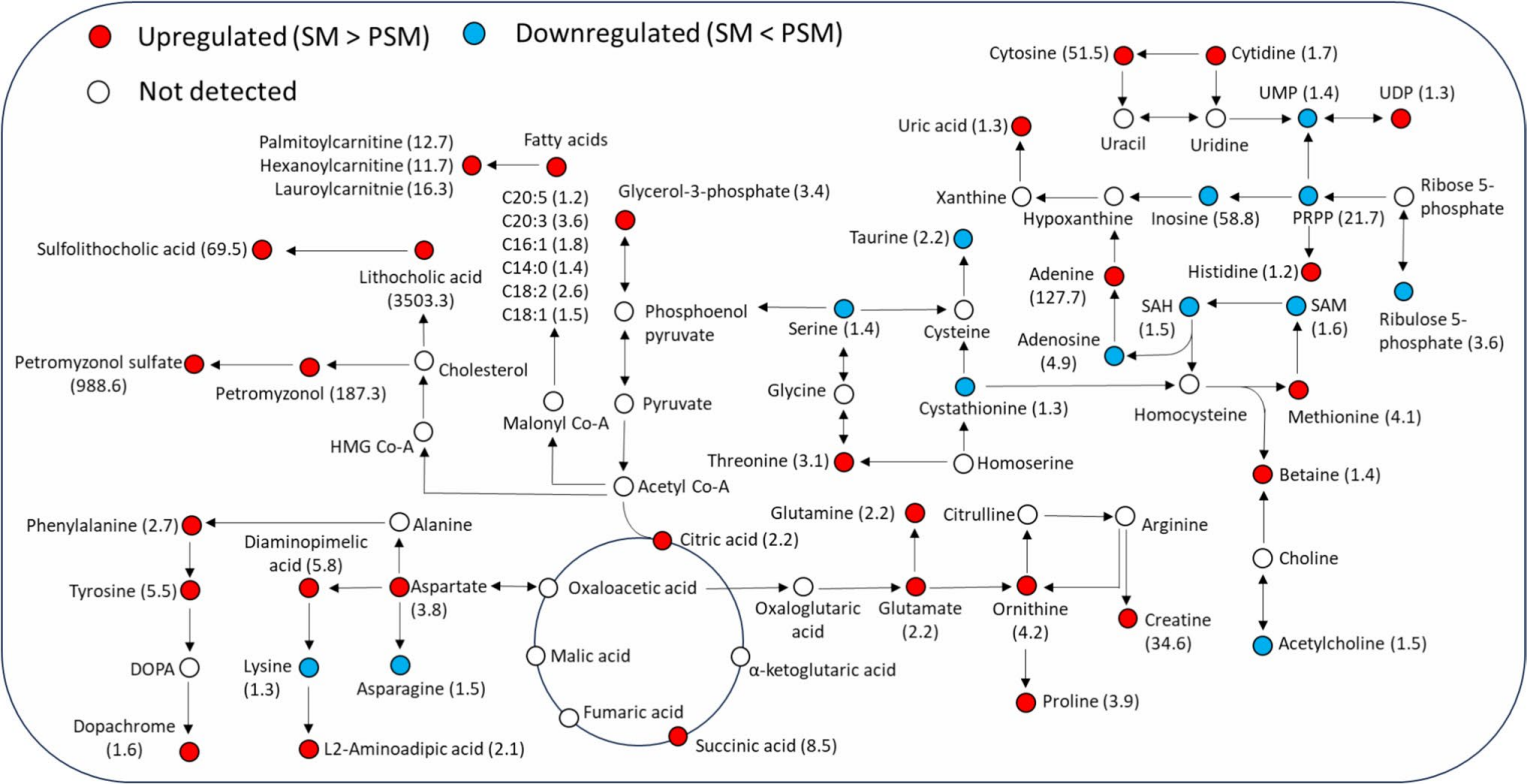
A simplified metabolic pathway map highlighting the compounds that were upregulated (red) or downregulated (blue) between spermiating males (SM) and prespemiating males (PSM). The numbers in the brackets represent the fold change between groups (Color figure online)

An overall upregulation of metabolic pathways was seen in the mature males compared to the immature ones. A simplified metabolic map representing the major pathways and highlighting regulation of the annotated metabolites is illustrated in Fig. 2. A complete list of metabolites with their fold change (FC) values, p-values, and q-values is provided as supplementary information in Table S1. A major upregulation of the secondary bile acid synthesis, lithocholic acid with an FC of 3503.3, was observed in SM. A comparatively modest level of increase was also seen in the sulfated form of the compound (sulfolithocholic acid) with an FC of 69.5. Sea lamprey specific bile acids such as petromyzonol sulfate (FC: 988.6), and petromyzonol (FC: 187.3) also showed a significant upregulation in SM. Several amino acids like tyrosine (FC: 5.5), phenylalanine (FC: 2.7), ornithine (FC: 4.2), methionine (FC: 4.1), proline (FC: 3.9) and their metabolic intermediates were upregulated in SM. However, slight downregulation was seen for amino acids like taurine (FC: 2.2), asparagine (FC: 1.5), serine (FC: 1.4), and lysine (FC: 1.3). Metabolites involved in the TCA cycle such as glycerol-3-phosphate (FC: 3.4), citric acid (FC: 2.2), and succinic acid (8.5) were all upregulated signifying increased levels of carbohydrate metabolism in SM compared to PSM (Fig. S2). Moreover, metabolites related to the pentose phosphate pathway such as phosphoribosyl pyrophosphate (FC: 21.74), and ribulose-5-phosphate (FC: 3.6) showed a significant downregulation in SM. The nucleotides adenine (FC: 127.7) and cytosine (FC: 51.5) showed major upregulation in SM. Similarly, an upregulation was seen for several fatty acids including linoleic acid (FC: 2.6), palmitoleic acid (FC: 1.8), oleic acid (FC: 1.5), and myristic acid (FC: 1.4); and for the fatty acyl carnitines like lauroylcarnitine (FC: 16.3), palmitoylcarnitine (FC: 12.7), and hexanoylcarnitine (FC: 11.7) in SM.

#### Preovulatory females versus ovulatory females

**Fig. 3:**
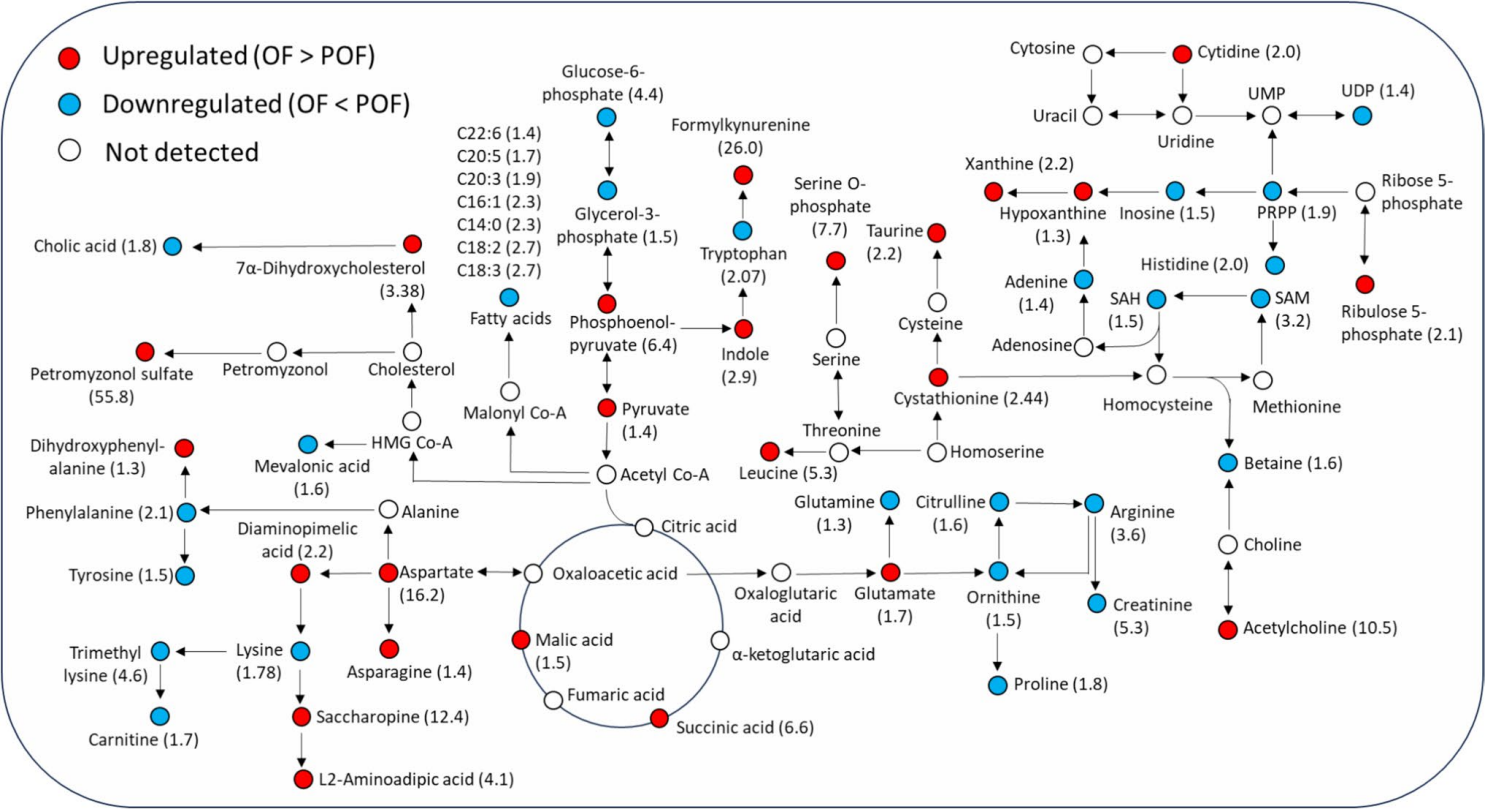
A simplified metabolic pathway map highlighting the compounds that were upregulated (red) or downregulated (blue) between ovulatory females (OF) and preovulatory females (POF). The numbers in the brackets represent the fold change between the two groups (Color figure online)

The levels of changes between the immature and mature females were less dramatic than their male counterparts. The major metabolites driving the changes between groups are mapped in a simplified metabolic map in Fig. 3. A complete list of metabolites with their FC values, p-values, and q-values is provided as supplementary information in Table S2. Like the male groups, significant upregulation in OF was seen for the sea lamprey specific bile acid, petromyzonol sulfate (FC: 55.8). In metabolites related to amino acid metabolism, the highest upregulation was seen for formylkynurenine (FC: 26.0), aspartic acid (FC: 16.2), and saccharopine (FC: 12.4) in OF. Other amino acids, including arginine (FC: 3.6), tryptophan (FC: 2.1), phenylalanine (FC: 2.1), histidine (FC: 2.0), lysine (FC: 1.8), and proline (FC: 1.8) indicated an overall downregulation of amino acid metabolism in OF (Fig. S3). Although some intermediates in the TCA cycle such as phosphoenolpyruvic acid (FC: 6.4), pyruvic acid (FC: 1.4), succinic acid (FC: 6.6), and malic acid (FC: 1.5) were upregulated; glucose-6-phosphate (FC: 4.4), 3-Phosphoglyceric acid (FC: 1.5), and lactic acid (FC: 7.0) were downregulated in OF. Similarly, in the pentose phosphate pathway, phosphoribosyl pyrophosphate (FC: 1.9), and ribulose diphosphate (FC: 1.3) were downregulated whereas ribulose-5-phosphate (FC: 2.1) was upregulated in OF. The most significantly upregulated metabolite related to nucleotide metabolism was uric acid (FC: 8.6) in OF. The most prominent change between POF and OF compared to the male groups was the overall downregulation of fatty acid metabolism (Fig. S3). Fatty acids including linolenic acid (FC: 2.7), linoleic acid (FC: 2.7), myristic acid (FC: 2.3), and palmitoleic acid (FC: 2.3) were all downregulated in OF. Moreover, metabolites involved in steroid biosynthesis, mevalonic acid (FC: 1.6) was also downregulated in OF.

#### Sex differences

The males and females were compared for their levels of changes in metabolites in both mature (OF vs. SM) and immature (POF vs. PSM) groups. The list of compounds with their FC values, p-values, and q-values is provided as supplementary information in Table S3 and S4. In the OF vs SM group, several amino acid metabolites were upregulated in OF, showing higher levels of saccharopine (FC: 26.3), glutamine (13.5), and indole (9.1) than SM. Comparatively, metabolites related to carbohydrate and fatty acid metabolism showed an overall upregulation in SM compared to OF (Fig. S4). All the annotated fatty acid metabolites were upregulated in SM with the FC values ranging from 1.2 to 2.8. Among the metabolites related to nucleotide metabolism, cytosine showed the highest upregulation in SM (FC: 34.8), and adenosine showed the highest downregulation in OF (FC: 3.9).

The levels of changes were slightly subdued in the immature groups compared to the mature groups. An overall downregulation was seen in the amino acid metabolism in PSM, with only taurine (FC: 3.2) and argininosuccinic acid (FC: 1.2) showing upregulation among all other annotated metabolites. Similarly, metabolites involved in carbohydrate metabolism and fatty acid metabolism were downregulated in POF. Cytidine (FC: 2.7), cytosine (FC: 2.6), and adenosine (FC: 1.6) related to nucleotide metabolism were upregulated in PSM.

### Sex and life stage differences in liver glycogen and triglyceride concentrations

**Fig. 4:**
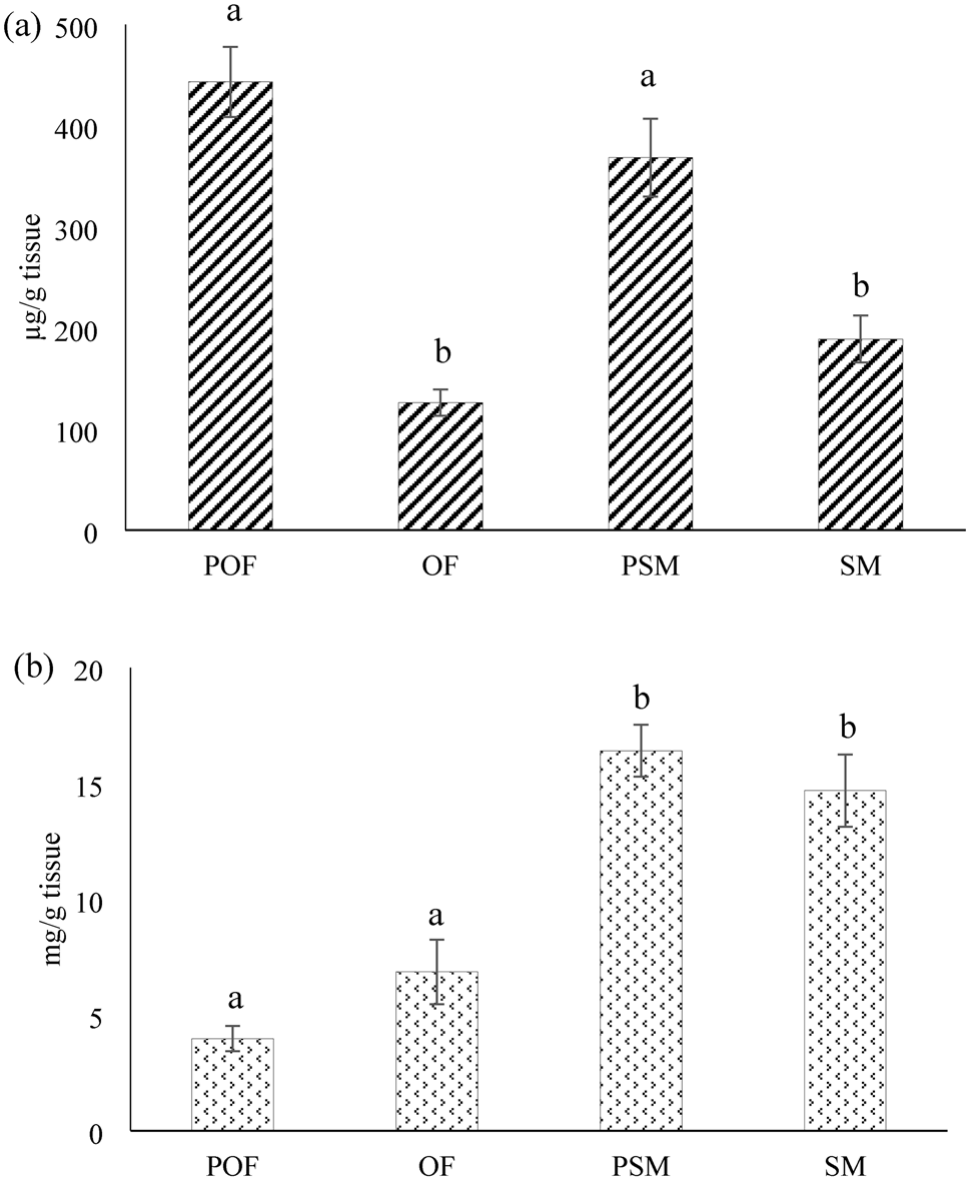
The glycogen concentrations (**a**) and the triglyceride concentrations (**b**) in preovulatory females (POF), ovulatory females (OF), prespermiating males (PSM), and spermiating males (SM)**.** The different letters above the bars represent significant differences between groups (p ≤ 0.0001)

Sexually immature sea lamprey had more liver glycogen than sexually mature ones as shown in Fig. 4a. ANOVA test showed that liver glycogen concentrations were significantly different among POF, OF, PSM, and SM (p < 0.0001, DF = 3). Post hoc tests showed that POF liver glycogen concentration (443.487 ± 34.762 µg/g tissue) was different from that of OF (126.253 ± 13.105 µg/g tissue; p < 0.0001) and SM (189.126 ± 23.140 µg/g tissue; p < 0.0001). PSM liver glycogen concentration (368.533 ± 38.470 µg/g tissue) was also significantly different from that of OF (p < 0.0001) and SM (p = 0.0001).

Interestingly, male sea lamprey had more liver triglyceride than females. The results are illustrated in Fig. 4b. ANOVA test showed that liver triglyceride concentrations were significantly different among POF, OF, PSM and SM (p < 0.0001, DF = 3). Post hoc tests showed that POF liver triglyceride concentration (3.978 ± 0.543 mg/g tissue) was significantly different from that of PSM (16.410 ± 1.119 mg/g tissue; p < 0.0001) and SM (14.688 ± 1.561 mg/g tissue; p < 0.0001). OF liver triglyceride concentration (6.859 ± 1.390 mg/g tissue) was also significantly different from that of PSM (p < 0.0001) and SM (p < 0.0001).

## Discussion

Adult sea lampreys undergo a starvation phase during migration until they die after spawning. During this period, carbohydrates, lipids, and proteins must be mobilized from muscles and other organs to meet the energy demands of long distance movement and growth of the gonads, especially formation of yolk material in females (Hardisty and Potter 1971, 1972; Moore & Potter, 1976). Glycogens and triglycerides are the primary carbohydrate and lipid storage molecules in the liver. Previous studies have shown that they play critical roles in lamprey liver during spawning migration (Bentley & Follett, 1965; Heikkala et al., 1984; Kott 1970). We further speculated that these molecules are essential fuels for the long-distance migration and rigorous spawning behaviors in adult lamprey. Triglycerides, when metabolized into free fatty acids and subsequently into acetyl CoA, are building blocks for cholesterol, the precursor for bile acids, and function as fuel through mitochondrial oxidative phosphorylation and respiratory chain.

It is known that river lampreys maintain normal blood glucose levels until shortly before death. However, at the time of ovulation or spermiation, the glucose levels rise (Larsen, 1969, 1976). The decrease in liver glycogen in mature sea lamprey compared to immature individuals indicates that liver glycogen may serve as a major fuel during migration. Indeed, previous reports have also shown decreased liver glycogen in lampreys during migration (Beamish et al., 1979; Bentley & Follett, 1965).

Our results indicate that SM exhibits up-regulated carbohydrate and lipid metabolisms as well as dynamic changes in amino acid and nucleotide metabolisms compared to PSM. On the other hand, OF exhibits up-regulated arachidonic acid (potential inducer for vitellogenesis) (Swetha et al., 2020; Wu et al., 2022), with dynamic changes in other metabolic pathways. The higher liver triglyceride concentrations in males confirmed that upregulation of lipid metabolism resulted in lipid accumulations in liver. Previous reports also showed dynamic changes in liver amino acid, carbohydrate, lipid, and protein concentrations in lamprey at different life stages (Beamish et al., 1979; Bentley & Follett, 1965; Wilkie et al., 2006). These results may offer insight into how males manage to increase pheromone production, whereas females ramp up vitellogenesis synthesis to support fecundity. Our previous studies showed that sea lamprey livers in both sexes produce bile acids, but they differ significantly in the amount of bile acids produced (Chung-Davidson et al., 2021). We also demonstrated that males drastically upregulate the rate-limiting enzyme for bile acid synthesis (CYP7A1) when they are sexually mature (Yeh et al., 2012). The upregulation of primary and secondary bile acids in spermiating males was also evident in the plasma metabolomes in a previous study [8].

Vitellogenin is a glycolipophosphoprotein containing 79% protein and 19% lipids, with 70% phospholipids in the rainbow trout (Fremont & Riazi, 1988). It is synthesized in the liver in response to estrogenic stimulation (Emmersen et al. 1976; Vaillant et al., 1988; van Bohemen et al., 1981). After leaving the bloodstream, vitellogenin goes through the follicle wall and is selectively incorporated into the oocyte by receptor-mediated endocytosis (Campbell et al. 1979; Davail et al., 1998; Rodriguez et al., 1996). Vitellogenin is then transferred into multivesicular bodies from the lysosomal compartment (Busson-Mabillot, 1984) where it is co-localized with a proteolytic enzyme, cathepsin D that cleaves it into yolk proteins (Sire et al., 1994). Lamprey vitellogenin was first cloned by Sharrock et al. (Sharrock et al., 1992) and showed similar response to estradiol stimulation (Mewes et al., 2002) Vitellogenesis is low in winter and increases rapidly in the last month before ovulation (Larsen, 1969). It is interesting that sea lamprey liver supplies lipoprotein vitellogenin to developing oocytes even though liver lipids decreased during migration (Arau´jo et al., 2013) and later maturation stages (Youson, 1981). Vitellogenesis requires an important metabolic effort from the female fish because ovary weight can reach up to 20 to 25% of body weight at the end of vitellogenesis (Jalabert, 2005). In general, the metabolites, including amino acids, are lower in ovulatory females in comparison to pre-ovulatory females, which is consistent with the notion that the vitellogenesis process ends about one month before the onset of ovulation (Clemens et al., 2013; Heikkala et al., 1984). The pre-ovulatory female used in this study was captured in the early season when the females entered the riverine phase of their reproductive migration and hence are likely still in the process of generating vitellogenin in their liver.

It is curious how lipid, protein and carbohydrate metabolism contribute to generating the ATP required by sea lamprey during the non-trophic upstream spawning migration. Energy is required for maintenance, swimming, the development of gonads and secondary sexual characters and spawning and post-spawning activities. It has been shown that energy is supplied predominantly via anaerobic metabolism in early migrants, and by aerobic metabolism in mature males and ovulatory females (Paton et al., 2020). Our results support the notion that mature sea lamprey generate ATP via aerobic metabolism, as the TCA cycle was upregulated in SM compared to PSM and in OF compared to POF. In particular, the key metabolites in TCA cycles were up-regulated in SM (compared to PSM), whereas phosphoenopyruvic acid (aerobic metabolite) was up-regulated and lactic acid (anaerobic metabolite) was down-regulated in OF (compared to POF). In conclusion, female and male sea lamprey adopt distinct metabolic strategies during sexual maturation.

## Supplementary Information

Below is the link to the electronic supplementary material.

Supplementary file1 (DOCX 9746 KB)

## Data Availability

The metabolomics and metadata reported in this paper have been deposited to the GNPS MassIVE repository at 10.25345/C5FQ9QH8G under MSV000096840.
