# Supplementary material for: Liver metabolomic profiles of sea lamprey (Petromyzon marinus) are influenced by sex and maturation stages
Source: Metabolomics. 2025 May 17;21(3):69. doi: 10.1007/s11306-025-02266-8 (PMC12085385; doi:10.1007/s11306-025-02266-8)
Supplement: Supplementary file 1 — Supplementary file1 (DOCX 9746 KB) [file 11306_2025_2266_MOESM1_ESM.docx]

Table S1. Metabolic pathways and the annotated metabolites with the most discriminant changes between pre-spermiating males (PSM) and spermiating males (SM). Upregulation means SM > PSM, and downregulation means PSM > SM.

| **Metabolism pathway** | **Metabolite** | **Regulation** | **Fold Change (max)** | **p-value** | **q-value** |
| --- | --- | --- | --- | --- | --- |
| Amino acid metabolism | Isovaleryl-CoA | Up | 35.01 | 2.49E-09 | 3.94E-07 |
|  | Creatine | Up | 34.56 | 1.15E-06 | 3.56E-05 |
|  | Reduced Glutathione | Up | 13.60 | 2.40E-04 | 1.69E-03 |
|  | 2,6-Diaminopimelic acid | Up | 5.78 | 4.35E-06 | 8.50E-05 |
|  | Tyrosine | Up | 5.53 | 4.05E-06 | 8.11E-05 |
|  | Ornithine | Up | 4.20 | 2.19E-03 | 9.19E-03 |
|  | Methionine | Up | 4.12 | 4.57E-06 | 1.10E-04 |
|  | Proline | Up | 3.89 | 8.26E-05 | 7.44E-04 |
|  | Isoleucine | Up | 3.89 | 2.13E-07 | 1.06E-05 |
|  | Aspartic acid | Up | 3.81 | 1.56E-04 | 1.21E-03 |
|  | Indole | Up | 3.65 | 3.39E-04 | 2.18E-03 |
|  | Threonine | Up | 3.10 | 4.72E-04 | 3.22E-03 |
|  | Pantothenic acid | Up | 3.06 | 1.98E-04 | 1.47E-03 |
|  | Phenylalanine | Up | 2.69 | 3.73E-02 | 8.68E-02 |
|  | 4-Guanidinobutyric acid | Down | 2.67 | 8.19E-02 | 1.52E-01 |
|  | Tryptophan | Up | 2.56 | 1.25E-05 | 1.81E-04 |
|  | Taurine | Down | 2.24 | 3.28E-02 | 7.85E-02 |
|  | Pyroglutamic acid | Up | 2.23 | 2.57E-05 | 3.95E-04 |
|  | Glutamine | Up | 2.17 | 2.54E-02 | 6.36E-02 |
|  | α-Aminoadipic acid | Up | 2.12 | 6.50E-02 | 1.27E-01 |
|  | Carnitine | Up | 1.92 | 8.55E-02 | 1.57E-01 |
|  | S-Adenosylmethionine | Down | 1.60 | 3.01E-01 | 8.40E-02 |
|  | Dopachrome | Up | 1.57 | 9.66E-04 | 8.90E-04 |
|  | S-Adenosylhomocysteine | Down | 1.48 | 9.08E-01 | 9.40E-01 |
|  | Dehydroascorbic acid | Up | 1.46 | 7.49E-03 | 2.37E-02 |
|  | Asparagine | Down | 1.46 | 1.86E-01 | 2.97E-01 |
|  | Serine | Down | 1.42 | 3.40E-01 | 4.59E-01 |
|  | Glutamic acid | Up | 1.38 | 9.11E-03 | 2.75E-02 |
|  | Betaine | Up | 1.38 | 3.80E-02 | 8.35E-02 |
|  | Lysine | Down | 1.34 | 2.11E-01 | 3.28E-01 |
|  | Formiminoglutamic Acid | Down | 1.30 | 7.26E-01 | 1.63E-01 |
|  | Cystathionine | Down | 1.26 | 6.69E-01 | 1.53E-01 |
|  | Adenylthiomethylpentose | Down | 1.25 | 3.38E-01 | 4.65E-01 |
|  | Histidine | Up | 1.22 | 1.58E-01 | 2.54E-01 |
|  |  |  |  |  |  |
| Dipeptides | Pro-Lys | Down | 349.01 | 1.70E-02 | 8.65E-03 |
|  | Homoanserine | Down | 59.98 | 4.79E-02 | 1.96E-02 |
|  | Ala-Pro | Down | 31.20 | 2.79E-07 | 9.38E-07 |
|  | glu-leu | Up | 8.92 | 6.06E-05 | 5.74E-05 |
|  | leu-lys | Up | 2.68 | 5.30E-04 | 5.46E-04 |
|  | asn-pro | Down | 2.26 | 4.22E-01 | 7.90E-02 |
|  |  |  |  |  |  |
| Carbohydrate metabolism | Succinic acid | Up | 8.47 | 7.74E-09 | 9.48E-07 |
|  | β-fucose 1-phosphate | Up | 6.59 | 4.62E-05 | 6.07E-04 |
|  | 3-sulfolactic acid | Up | 6.30 | 1.40E-05 | 2.53E-04 |
|  | Adenosine diphosphate (ADP) | Up | 2.42 | 4.48E-02 | 9.48E-02 |
|  | Citric acid | Up | 2.15 | 4.98E-03 | 1.72E-02 |
|  | Adenosine triphosphate (ATP) | Up | 1.81 | 7.04E-01 | 7.85E-01 |
|  | 3-Phosphoglyceric acid | Up | 1.77 | 3.67E-01 | 4.86E-01 |
|  |  |  |  |  |  |
| Pentose phosphate pathway | Phosphoribosyl pyrophosphate | Down | 21.74 | 1.03E-03 | 5.67E-03 |
|  | ribulose 5-phosphate | Down | 3.57 | 5.17E-07 | 2.39E-05 |
|  | Deoxyribose | Down | 3.51 | 5.93E-06 | 1.31E-05 |
|  | Gluconic acid | Up | 1.48 | 3.76E-02 | 8.72E-02 |
|  |  |  |  |  |  |
| Nucleotide metabolism | Adenine | Up | 127.71 | 2.42E-12 | 1.07E-09 |
|  | Inosine | Down | 58.82 | 5.12E-15 | 3.56E-12 |
|  | Cytosine | Up | 51.50 | 1.76E-13 | 9.52E-11 |
|  | Orotic acid | Up | 31.17 | 7.18E-08 | 5.58E-06 |
|  | Adenosine | Down | 4.93 | 1.36E-03 | 6.33E-03 |
|  | Cytidine | Up | 1.72 | 1.45E-02 | 3.92E-02 |
|  | Adenylsuccinic acid | Up | 1.63 | 2.40E-01 | 3.51E-01 |
|  | Uridine monophosphate | Down | 1.35 | 4.76E-02 | 9.96E-02 |
|  | Guanosine monophosphate | Up | 1.34 | 1.26E-01 | 2.13E-01 |
|  | uridine 5′-diphosphate | Up | 1.32 | 4.26E-01 | 5.49E-01 |
|  | Uric Acid | Up | 1.26 | 2.05E-01 | 3.21E-01 |
|  |  |  |  |  |  |
| Fatty acid metabolism | Lauroylcarnitine | Up | 16.28 | 1.02E-04 | 1.42E-04 |
|  | Palmitoylcarnitine | Up | 12.66 | 5.32E-06 | 9.78E-05 |
|  | Hexanoylcarnitine | Up | 11.69 | 1.10E-05 | 1.67E-04 |
|  | Dihomo-γ-Linolenic Acid | Up | 3.63 | 4.31E-03 | 1.66E-02 |
|  | Glycerol 3-phosphate | Up | 3.40 | 9.67E-05 | 8.35E-04 |
|  | Linoleic Acid | Up | 2.55 | 1.41E-02 | 4.09E-02 |
|  | Acetyl-carnitine | Up | 2.30 | 1.45E-01 | 2.37E-01 |
|  | Palmitoleic Acid | Up | 1.81 | 3.86E-01 | 5.12E-01 |
|  | Oleic Acid | Up | 1.54 | 2.13E-01 | 3.30E-01 |
|  | Acetylcholine | Down | 1.49 | 4.63E-03 | 1.63E-02 |
|  | Myristic Acid | Up | 1.42 | 4.96E-01 | 6.12E-01 |
|  | (±)18-HEPE | Up | 1.40 | 2.98E-01 | 4.22E-01 |
|  | Eicosapentanoic acid | Up | 1.22 | 9.64E-01 | 9.75E-01 |
|  |  |  |  |  |  |
| Steroid hormone biosynthesis | Testosterone | Up | 1.39 | 8.87E-01 | 9.21E-01 |
| Secondary bile acid biosynthesis | Lithocholic acid | Up | 3503.30 | 1.87E-05 | 3.42E-05 |
|  | Muricholic Acid | Up | 13.09 | 1.68E-04 | 1.51E-03 |
| Nicotinate & nicotinamide metabolism | Nicotinamide | Up | 3.15 | 1.36E-03 | 6.33E-03 |
| Riboflavin metabolism | Flavin mononucleotide (riboflavin-5'-phosphate) | Up | 2.57 | 7.75E-04 | 7.50E-04 |
| amino sugar and nucleotide sugar metabolism | N-Acetylneuraminic acid | Up | 2.40 | 1.30E-04 | 1.74E-04 |
| pentose and glucuronate interconversion | D-Xylonic acid | Down | 2.59 | 2.76E-05 | 2.93E-05 |
| Vitamin B6 metabolism | Pyridoxal | Down | 2.34 | 3.50E-01 | 4.76E-01 |
|  |  |  |  |  |  |
| Other pathways | Petromyzonol sulfate | Up | 988.56 | 2.26E-12 | 1.11E-09 |
|  | Petromyzonol | Up | 187.30 | 7.12E-08 | 2.84E-07 |
|  | Arabinosylhypoxanthine | Down | 100.00 | 1.37E-10 | 3.52E-08 |
|  | Sulfolithocholic acid | Up | 69.49 | 6.91E-12 | 6.13E-11 |
|  | N-acetylleukotriene E4 | Up | 50.63 | 2.14E-05 | 2.34E-05 |
|  | 8-hydroxy-deoxyguanosine | Down | 34.48 | 1.61E-09 | 2.92E-07 |
|  | γ-Glutamylphenylalanine | Up | 12.06 | 1.82E-03 | 9.38E-04 |
|  | N3,N4-Dimethylarginine | Down | 5.35 | 2.01E-04 | 1.48E-03 |
|  | 4,6-Cholestadien-3-one | Down | 4.24 | 5.89E-02 | 1.18E-01 |
|  | Thyronine | Down | 4.02 | 7.54E-04 | 7.32E-04 |
|  | Hexose | Down | 2.50 | 2.71E-05 | 4.14E-04 |
|  | N-Acetylmethionine | Up | 2.41 | 3.73E-03 | 1.66E-03 |
|  | Cysteine-glutathione disulfide | Down | 1.77 | 4.15E-02 | 1.18E-02 |
|  | Aceturic acid (N-acetyl glycine) | Up | 1.72 | 1.92E-03 | 8.97E-03 |
|  | 9-riburonosyladenine | Up | 1.54 | 3.13E-03 | 2.28E-03 |
|  | 12-Hydroxylauric acid | Up | 1.51 | 1.64E-01 | 5.20E-02 |
|  | Lysophosphatidylinositol | Down | 1.50 | 2.02E-02 | 6.53E-03 |
|  | Hydroxystearic acid | Down | 1.41 | 1.37E-02 | 4.79E-03 |

Table S2. Metabolic pathways and the annotated metabolites with the most discriminant changes between pre-ovulating females (POF) and ovulating females (OF). Upregulation means OF > POF, and downregulation means POF > OF.

| **Metabolism pathway** | **Metabolite** | **Regulation** | **Fold Change (max)** | **p-value** | **q-value** |
| --- | --- | --- | --- | --- | --- |
| Amino acid metabolism | Formylkynurenine | Up | 26.01 | 4.94E-07 | 5.08E-06 |
|  | Aspartic acid | Up | 16.23 | 7.81E-05 | 5.46E-04 |
|  | Saccharopine | Up | 12.37 | 2.13E-07 | 7.02E-06 |
|  | Serine O-phosphate | Up | 7.65 | 6.20E-01 | 7.25E-01 |
|  | Creatinine | Down | 5.32 | 5.02E-04 | 2.41E-03 |
|  | Leucine | Up | 5.26 | 3.28E-09 | 3.07E-07 |
|  | N6,N6,N6-Trimethyllysine | Down | 4.57 | 7.11E-07 | 1.75E-05 |
|  | 2-Aminoadipic acid | Up | 4.08 | 8.85E-06 | 9.84E-05 |
|  | Arginine | Down | 3.64 | 9.74E-03 | 2.74E-02 |
|  | Reduced Glutathione | Up | 3.61 | 2.61E-02 | 6.19E-02 |
|  | S-Adenosyl-methionine | Down | 3.23 | 3.42E-07 | 3.78E-06 |
|  | Indole | Up | 2.86 | 3.08E-03 | 1.05E-02 |
|  | N(2)-succinyl-glutamic acid | Down | 2.76 | 8.75E-04 | 1.03E-03 |
|  | Pantothenic acid | Down | 2.56 | 1.24E-09 | 6.68E-09 |
|  | Dehydroascorbic acid | Up | 2.49 | 8.44E-02 | 1.40E-01 |
|  | Cystathionine | Up | 2.44 | 9.98E-02 | 1.84E-01 |
|  | Taurine | Up | 2.24 | 1.31E-02 | 3.48E-02 |
|  | 2,6-Diaminopimelic acid | Up | 2.20 | 2.20E-02 | 4.39E-02 |
|  | Tryptophan | Down | 2.07 | 5.68E-05 | 9.16E-05 |
|  | Phenylalanine | Down | 2.05 | 2.60E-04 | 1.42E-03 |
|  | Histidine | Down | 1.96 | 1.21E-03 | 4.89E-03 |
|  | Lysine | Down | 1.78 | 1.65E-02 | 4.22E-02 |
|  | Proline | Down | 1.76 | 2.17E-02 | 5.32E-02 |
|  | Glutamic acid | Up | 1.73 | 1.09E-02 | 3.00E-02 |
|  | Pipecolinic acid | Down | 1.72 | 4.51E-02 | 9.66E-02 |
|  | Carnitine | Down | 1.66 | 3.28E-03 | 1.10E-02 |
|  | Betaine | Down | 1.59 | 7.65E-01 | 8.40E-01 |
|  | Lysine | Down | 1.58 | 6.31E-04 | 2.03E-03 |
|  | Citrulline | Down | 1.56 | 2.96E-01 | 3.97E-01 |
|  | Ornithine | Down | 1.47 | 2.72E-01 | 3.98E-01 |
|  | S-Adenosylhomocysteine | Down | 1.46 | 1.29E-01 | 2.25E-01 |
|  | Tyrosine | Down | 1.45 | 1.03E-01 | 7.40E-02 |
|  | Pyroglutamic Acid | Up | 1.38 | 4.69E-02 | 1.00E-01 |
|  | 5,6-dihydroxy-2-indolecarboxylic acid | Up | 1.37 | 6.03E-02 | 1.23E-01 |
|  | Asparagine | Up | 1.36 | 6.30E-01 | 5.00E-01 |
|  | α-ketoadipic acid | Up | 1.34 | 3.49E-01 | 4.52E-01 |
|  | Glutamine | Down | 1.34 | 4.14E-02 | 3.25E-02 |
|  | Dihydroxyphenylalanine | Up | 1.34 | 7.38E-04 | 2.33E-03 |
|  | Phosphoserine | Down | 1.25 | 3.10E-01 | 1.97E-01 |
|  | γ-Aminobutyric acid | Up | 1.23 | 3.25E-01 | 4.55E-01 |
|  |  |  |  |  |  |
| Dipeptides | Gly-Lys | Down | 15.29 | 1.81E-03 | 4.00E-03 |
|  | Pro-Hyp | Up | 12.37 | 7.10E-10 | 4.03E-09 |
|  | 4-Hydroxyprolyllysine | Down | 3.88 | 1.39E-05 | 7.73E-05 |
|  | Tyr-Gly | Up | 2.79 | 5.75E-03 | 1.03E-02 |
|  | Isoleucyl-4-hydroxyproline | Down | 2.03 | 1.19E-02 | 1.88E-02 |
|  | Tyr-Trp | Down | 1.83 | 1.57E-02 | 2.35E-02 |
|  | Phe-Lys | Up | 1.81 | 4.65E-02 | 5.80E-02 |
|  | Ile-Leu | Up | 1.59 | 1.52E-01 | 1.04E-01 |
|  | β-Ala-Lys | Up | 1.58 | 3.79E-01 | 2.34E-01 |
|  | Thr-Ile | Up | 1.40 | 6.86E-01 | 3.91E-01 |
|  | γ -Glutamyl-L-glutamic acid | Up | 1.37 | 1.38E-01 | 2.37E-01 |
|  | Gly-Leu | Up | 1.26 | 5.07E-01 | 6.25E-01 |
|  |  |  |  |  |  |
| Carbohydrate metabolism | Phosphoenolpyruvic acid | Up | 6.35 | 6.53E-01 | 7.48E-01 |
|  | Lactic Acid | Down | 6.99 | 2.53E-10 | 1.43E-08 |
|  | Succinic acid | Up | 6.59 | 8.98E-09 | 2.49E-07 |
|  | Glucose 6-phosphate | Down | 4.41 | 1.79E-08 | 1.01E-06 |
|  | 2,3-Diphosphoglyceric acid | Down | 2.48 | 1.17E-03 | 4.76E-03 |
|  | 3-Phosphoglyceric acid | Down | 1.49 | 1.07E-01 | 1.94E-01 |
|  | Malic Acid | Up | 1.45 | 7.15E-03 | 1.67E-02 |
|  | Pyruvic acid | Up | 1.42 | 1.13E-02 | 2.47E-02 |
|  | Adenosine triphosphate | Down | 1.27 | 2.66E-01 | 3.63E-01 |
|  | Adenosine diphosphate | Down | 1.26 | 7.67E-01 | 8.28E-01 |
|  |  |  |  |  |  |
| Pentose phosphate pathway | Ribulose 5-phosphate | Up | 2.10 | 3.67E-05 | 1.83E-04 |
|  | Glucono-δ-lactone | Up | 1.28 | 1.97E-01 | 2.84E-01 |
|  | Phosphoribosyl pyrophosphate | Down | 1.86 | 7.74E-01 | 4.35E-01 |
|  | Gluconic acid | Down | 1.34 | 3.82E-01 | 4.86E-01 |
|  | Ribulose diphosphate | Down | 1.30 | 3.97E-01 | 5.01E-01 |
|  |  |  |  |  |  |
| Nucleotide metabolism | Uric Acid | Up | 8.58 | 8.23E-06 | 5.31E-05 |
|  | Guanosine triphosphate | Down | 4.58 | 1.12E-05 | 2.18E-05 |
|  | Uridine diphosphate glucose | Up | 2.76 | 1.38E-02 | 2.93E-02 |
|  | Xanthine | Up | 2.21 | 2.09E-03 | 5.80E-03 |
|  | Inosine-5'-monophosphate (IMP) | Up | 2.07 | 1.60E-01 | 2.66E-01 |
|  | Cytidine | Up | 1.96 | 3.46E-02 | 7.86E-02 |
|  | UDP-GlcNAc | Down | 1.53 | 3.84E-01 | 4.88E-01 |
|  | Inosine | Down | 1.45 | 8.22E-03 | 2.38E-02 |
|  | Phosphoribosyl pyrophosphate | Down | 1.45 | 8.49E-01 | 9.03E-01 |
|  | uridine 5′-diphosphate | Down | 1.44 | 6.03E-01 | 6.89E-01 |
|  | Adenylsuccinic acid | Up | 1.41 | 3.22E-01 | 4.52E-01 |
|  | Adenine | Down | 1.36 | 5.67E-01 | 6.80E-01 |
|  | Galacturonate-1-phosphate | Down | 1.31 | 1.78E-01 | 2.89E-01 |
|  | Hypoxanthine | Up | 1.29 | 2.46E-01 | 3.67E-01 |
|  |  |  |  |  |  |
| Fatty acid metabolism | Propionylcarnitine | Up | 4.02 | 5.88E-01 | 6.96E-01 |
|  | Linolenic Acid | Down | 2.73 | 4.32E-04 | 1.48E-03 |
|  | Linoleic Acid | Down | 2.67 | 5.51E-04 | 1.82E-03 |
|  | Myristic Acid | Down | 2.29 | 1.07E-04 | 4.46E-04 |
|  | Palmitoleic Acid | Down | 2.27 | 6.09E-04 | 1.98E-03 |
|  | Dihomo- γ -Linolenic Acid | Down | 1.86 | 6.64E-02 | 1.14E-01 |
|  | Eicosapentanoic acid | Down | 1.70 | 5.59E-01 | 6.53E-01 |
|  | Docosahexaenoic Acid | Down | 1.43 | 2.42E-01 | 3.36E-01 |
|  | Acetylcarnitine | Down | 1.40 | 7.61E-02 | 1.48E-01 |
|  | Arachidonic acid | Up | 1.31 | 4.88E-01 | 5.92E-01 |
|  | Dodecanedioic acid | Up | 1.27 | 8.89E-01 | 4.92E-01 |
|  |  |  |  |  |  |
| Phospholipid biosynthesis | Acetylcholine | Up | 10.50 | 3.20E-04 | 1.69E-03 |
|  | Glycerophosphorylethanolamine | Down | 6.73 | 0.00E+00 | 0.00E+00 |
| Steroid biosynthesis | 7-Dehydrodesmosterol | Up | 3.78 | 1.68E-08 | 3.24E-07 |
|  | Mevalonic acid | Down | 1.58 | 4.60E-01 | 5.64E-01 |
|  | Pregnane-3,20-dione | Down | 1.59 | 1.08E-01 | 1.73E-01 |
| Primary Bile acid biosynthesis | 7α,25-Dihydroxycholesterol | Up | 3.38 | 9.58E-06 | 1.03E-04 |
|  | Cholic acid | Down | 1.76 | 1.28E-08 | 5.45E-08 |
| Phosphonate metabolism | Phosphonoacetate | Up | 1.31 | 1.34E-01 | 2.06E-01 |
| Vitamin B6 metabolism | Pyridoxal | Down | 1.68 | 5.11E-04 | 1.71E-03 |
|  |  |  |  |  |  |
| Other pathways | Petromyzonol sulfate | Up | 55.77 | 4.44E-07 | 5.32E-06 |
|  | Dihydrouridine | Up | 43.52 | 1.40E-05 | 7.78E-05 |
|  | N(1)-acetylspermidine | Up | 17.65 | 8.55E-15 | 1.55E-12 |
|  | 1-Myristoyl-sn-glycerol 3-phosphate | Down | 12.37 | 3.44E-01 | 3.06E-01 |
|  | P-DMEA | Down | 10.00 | 1.50E-06 | 1.38E-05 |
|  | 5'-5,6-Dihydrouridylic acid | Up | 9.18 | 1.44E-07 | 1.85E-06 |
|  | Homocysteic acid | Up | 7.31 | 3.24E-03 | 3.32E-03 |
|  | N3,N4-Dimethylarginine | Down | 6.54 | 2.88E-06 | 4.66E-05 |
|  | α-Glycerylphosphorylcholine | Down | 5.32 | 5.29E-09 | 1.31E-07 |
|  | 4,6-Cholestadien-3-one | Up | 5.30 | 6.65E-06 | 8.04E-05 |
|  | 3-Hydroxyphenylglycine | Up | 5.02 | 2.22E-05 | 1.13E-04 |
|  | 3-hydroxytetradecanoylcarnitine | Up | 2.90 | 3.03E-03 | 6.09E-03 |
|  | Tryptophan N-glucoside | Up | 2.27 | 2.04E-06 | 1.65E-05 |
|  | Aceturic acid (N-acetylglycine) | Up | 1.97 | 1.28E-02 | 2.77E-02 |
|  | 3-hydroxyarachidonoylcarnitine | Down | 1.93 | 1.26E-02 | 1.96E-02 |
|  | 10-Hydroxyundecanoic acid | Up | 1.73 | 9.97E-05 | 3.81E-04 |
|  | Arabinosylhypoxanthine | Down | 1.69 | 1.84E-04 | 2.60E-04 |
|  | 10-hydroxystearic acid | Down | 1.52 | 4.58E-09 | 2.19E-08 |
|  | 4-Methyleneglutamine | Down | 1.49 | 3.86E-01 | 3.36E-01 |
|  | 8-hydroxy-deoxyguanosine | Down | 1.47 | 2.11E-02 | 5.19E-02 |
|  | 7-Methylguanine | Up | 1.23 | 1.41E-01 | 2.41E-01 |

Table S3. Metabolic pathways and the annotated metabolites with the most discriminant changes between spermiating males (SM) and ovulating females (OF). Upregulation means SM > OF, and downregulation means OF > SM.

| **Metabolism pathway** | **Metabolite** | **Regulation** | **Fold Change (max)** | **p-value** | **q-value** |
| --- | --- | --- | --- | --- | --- |
| Amino acid metabolism | Saccharopine | Down | 26.32 | 4.21E-07 | 8.57E-06 |
|  | Glutamine | Down | 13.48 | 1.72E-05 | 2.24E-05 |
|  | Indole | Down | 9.09 | 6.40E-03 | 2.73E-02 |
|  | 5'-S-Methyl-5'-thioadenosine | Up | 2.89 | 1.69E-02 | 5.91E-02 |
|  | Pantothenic acid | Up | 2.11 | 5.66E-02 | 1.92E-01 |
|  | Taurine | Down | 1.64 | 1.48E-01 | 3.15E-01 |
|  | Betaine | Up | 1.52 | 4.14E-01 | 6.06E-01 |
|  | Citrulline | Up | 1.41 | 1.43E-01 | 3.49E-01 |
|  | Glutamic acid | Down | 1.39 | 2.85E-01 | 4.91E-01 |
|  | Cystathionine | Down | 1.34 | 7.63E-01 | 8.74E-01 |
|  | 2,3,4,5-tetrahydrodipicolinic acid | Up | 1.20 | 4.88E-01 | 7.04E-01 |
|  |  |  |  |  |  |
| Carbohydrate metabolism | Adenosine triphosphate | Up | 4.19 | 9.54E-07 | 5.94E-07 |
|  | Adenosine diphosphate | Up | 3.43 | 1.70E-08 | 1.42E-08 |
|  | Glucose 6-phosphate | Up | 2.48 | 5.68E-04 | 3.54E-03 |
|  | 3-Phosphoglyceric acid | Up | 1.28 | 2.91E-01 | 5.36E-01 |
|  |  |  |  |  |  |
| Pentose phosphate pathway | Phosphoribosyl pyrophosphate | Down | 10.42 | 3.51E-05 | 8.39E-04 |
|  | Ribulose diphosphate | Down | 2.77 | 1.15E-02 | 5.63E-02 |
|  |  |  |  |  |  |
| Nucleotide metabolism | Cytosine | Up | 34.79 | 1.07E-10 | 5.49E-08 |
|  | Adenosine | Down | 3.94 | 4.67E-06 | 7.06E-05 |
|  | Cytidine | Down | 3.37 | 6.71E-03 | 2.82E-02 |
|  | 5-aminoimidazole ribotide | Up | 2.53 | 7.73E-02 | 1.98E-01 |
|  | Adenylsuccinic acid | Down | 1.49 | 6.54E-01 | 8.15E-01 |
|  | Hypoxanthine | Up | 1.31 | 9.58E-02 | 2.30E-01 |
|  | Uracil | Down | 1.28 | 1.61E-01 | 3.75E-01 |
|  |  |  |  |  |  |
| Fatty acid metabolism | Linolenic Acid | Up | 2.76 | 6.63E-02 | 2.11E-01 |
|  | Linoleic Acid | Up | 2.04 | 1.02E-01 | 2.81E-01 |
|  | Palmitoleic Acid | Up | 2.03 | 1.70E-01 | 3.92E-01 |
|  | Acetylcarnitine | Up | 2.03 | 5.59E-01 | 7.31E-01 |
|  | Docosahexaenoic Acid | Up | 1.95 | 2.63E-01 | 5.01E-01 |
|  | Glutaric acid | Up | 1.66 | 4.45E-01 | 6.65E-01 |
|  | Hexanoylcarnitine | Up | 1.45 | 7.60E-01 | 8.60E-01 |
|  | Arachidonic acid | Up | 1.30 | 6.46E-01 | 8.11E-01 |
|  | Oleic Acid | Up | 1.21 | 7.06E-01 | 8.39E-01 |
|  |  |  |  |  |  |
| Amino sugar metabolism | UDP-N-acetylglucosamine | Up | 1.57 | 9.03E-01 | 9.53E-01 |
| Amino sugar & nucleotide sugar metabolism | UDP-GlcNAc | Up | 1.30 | 8.58E-01 | 9.25E-01 |
| Nucleotide sugar metabolism | Uridine diphosphate glucose | Down | 1.41 | 2.91E-01 | 5.36E-01 |
| Benzoate degradation | Benzoic acid | Down | 1.92 | 1.25E-03 | 1.06E-02 |
|  |  |  |  |  |  |
| Other pathways | Docosatrienoic acid | Up | 3.12 | 4.66E-07 | 9.03E-07 |
|  | 2'-Deoxycytosine | Up | 2.06 | 2.46E-08 | 6.52E-08 |
|  | hydroxystearic acid | Up | 2.04 | 3.13E-11 | 4.38E-11 |
|  | 3-sulfolactic acid | Down | 1.73 | 6.73E-01 | 8.27E-01 |
|  |  |  |  |  |  |

Table S4. Metabolic pathways and the annotated metabolites with the most discriminant changes between pre-spermiating males (PSM) and pre-ovulating females (POF). Upregulation means PSM > POF, and downregulation means POF > PSM.

| **Metabolism pathway** | **Metabolite** | **Regulation** | **Fold Change (max)** | **p-value** | **q-value** |
| --- | --- | --- | --- | --- | --- |
| Amino acid metabolism | 2-Phenylalanine | Down | 5.35 | 1.41E-13 | 3.06E-13 |
|  | Tyrosine | Down | 5.20 | 2.97E-12 | 1.69E-11 |
|  | Homoserine | Down | 4.93 | 1.51E-12 | 2.74E-12 |
|  | Threonine | Down | 4.93 | 1.51E-12 | 2.74E-12 |
|  | Saccharopine | Down | 4.59 | 1.38E-04 | 1.06E-03 |
|  | Tryptophan | Down | 4.02 | 3.02E-11 | 4.23E-11 |
|  | Taurine | Up | 3.23 | 5.12E-03 | 2.00E-02 |
|  | 2-Aminoisobutyric Acid | Down | 2.16 | 3.00E-06 | 5.04E-06 |
|  | Methionine | Down | 2.06 | 1.51E-04 | 1.95E-03 |
|  | 5'-S-Methyl-5'-thioadenosine | Down | 1.80 | 1.03E-01 | 2.22E-01 |
|  | Pantothenic acid | Down | 1.46 | 2.11E-02 | 8.81E-02 |
|  | 5,6-dihydroxy-2-indolecarboxylic acid | Down | 1.46 | 1.81E-03 | 8.44E-03 |
|  | N,N-Dihydroxyvaline | Down | 1.45 | 4.03E-02 | 1.40E-01 |
|  | Betaine | Down | 1.35 | 6.76E-02 | 1.60E-01 |
|  | Argininosuccinic acid | Up | 1.22 | 5.65E-01 | 7.12E-01 |
|  |  |  |  |  |  |
| Dipeptides | Trp-Lys | Down | 18.54 | 4.65E-14 | 3.97E-13 |
|  | Phe-Thr | Down | 10.61 | 7.78E-05 | 3.97E-05 |
|  |  |  |  |  |  |
| Carbohydrate metabolism | Glycerol 3-phosphate | Down | 2.94 | 3.45E-05 | 3.33E-04 |
|  | 3-Phosphoglyceric acid | Down | 1.87 | 8.42E-02 | 2.32E-01 |
|  | Glucose 6-phosphate | Down | 1.79 | 4.80E-04 | 2.78E-03 |
|  | 2,3-Diphosphoglyceric acid | Down | 1.66 | 3.06E-02 | 8.83E-02 |
|  | Adenosine triphosphate | Down | 1.39 | 2.15E-01 | 4.37E-01 |
|  | Malic acid | Down | 1.39 | 5.75E-05 | 2.99E-05 |
|  |  |  |  |  |  |
| Pentose phosphate pathway | Phosphoribosyl pyrophosphate | Up | 1.89 | 4.19E-01 | 6.54E-01 |
|  | Ribulose diphosphate | Up | 1.71 | 7.16E-01 | 8.55E-01 |
|  | D-Gluconic acid | Down | 1.27 | 1.20E-01 | 2.97E-01 |
|  |  |  |  |  |  |
| Nucleotide metabolism | Cytidine | Up | 2.72 | 1.17E-02 | 3.90E-02 |
|  | Cytosine | Up | 2.55 | 3.79E-03 | 1.57E-02 |
|  | Guanosine diphosphate | Down | 1.61 | 4.88E-03 | 2.86E-02 |
|  | Adenosine | Up | 1.56 | 4.95E-01 | 6.56E-01 |
|  | Adenylsuccinic acid | Down | 1.47 | 5.79E-01 | 7.83E-01 |
|  | Hypoxanthin | Down | 1.37 | 1.45E-02 | 4.64E-02 |
|  |  |  |  |  |  |
| Fatty acid metabolism | Linoleic Acid | Down | 2.48 | 1.07E-02 | 5.39E-02 |
|  | Docosahexaenoic Acid | Down | 2.14 | 3.76E-02 | 1.33E-01 |
|  | Lauroylcarnitine | Down | 1.63 | 1.91E-02 | 1.59E-02 |
|  | Palmitoleic Acid | Down | 1.53 | 5.28E-02 | 1.69E-01 |
|  | Oleic Acid | Down | 1.35 | 6.81E-02 | 2.03E-01 |
|  |  |  |  |  |  |
| Amino sugar & nucleotide sugar metabolism | UDP-GlcNAc | Down | 2.26 | 7.55E-02 | 2.17E-01 |
| Amino sugar metabolism | N-Acetylneuraminic acid | Down | 2.03 | 2.34E-03 | 1.04E-02 |
| Benzoate degradation | Benzoic acid | Down | 5.75 | 6.64E-08 | 1.81E-06 |
|  |  |  |  |  |  |
| Other pathways | Saccharolactone | Up | 29.89 | 5.03E-06 | 8.12E-06 |
|  | Hydroxyacetone phosphate | Down | 2.48 | 5.01E-04 | 2.88E-03 |
|  | Diglycolic acid | Down | 1.39 | 5.75E-05 | 2.99E-05 |

Table S5. PCA and PLSDA fit and prediction parameters in the negative ionization mode

| Group | Plot | R2X (cum) | R2Y (cum) | Q2 (cum) |
| --- | --- | --- | --- | --- |
| PSM vs SM | PCA | 0.691 | - | 0.395 |
| POF vs OF | PCA | 0.638 | - | 0.444 |
| OF vs SM | PCA | 0.678 | - | 0.497 |
| POF vs PSM | PCA | 0.759 | - | 0.509 |
| PSM vs SM | PLS-DA | 0.614 | 0.991 | 0.968 |
| POF vs OF | PLS-DA | 0.605 | 0.987 | 0.952 |
| OF vs SM | PLS-DA | 0.957 | 0.904 | 0.904 |
| POF vs PSM | PLS-DA | 0.638 | 0.900 | 0.850 |

Figure S1. PLSDA score plots of the metabolic fingerprints of the liver extracts of sea lamprey in negative ionization mode: (a) prespermiating males (PSM) vs. spermiating males (SM); (b) preovulatory females (POF) vs. ovulatory females (OF); (c) OF vs SM; (d) POF vs PSM. The colors of the circles in the plot represent different sample groups: Light blue is PSM, purple is SM, yellow is POF, red is OF, green is female QC, and dark blue is male QC.

(a)


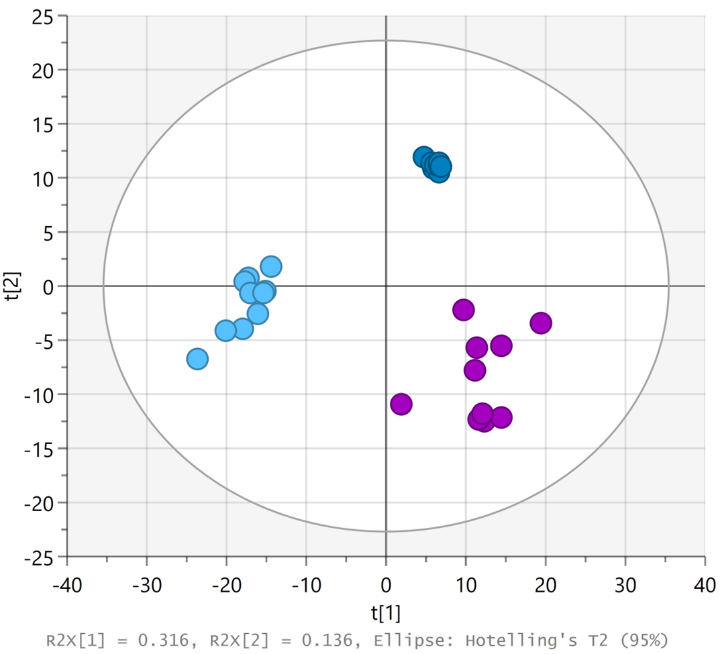

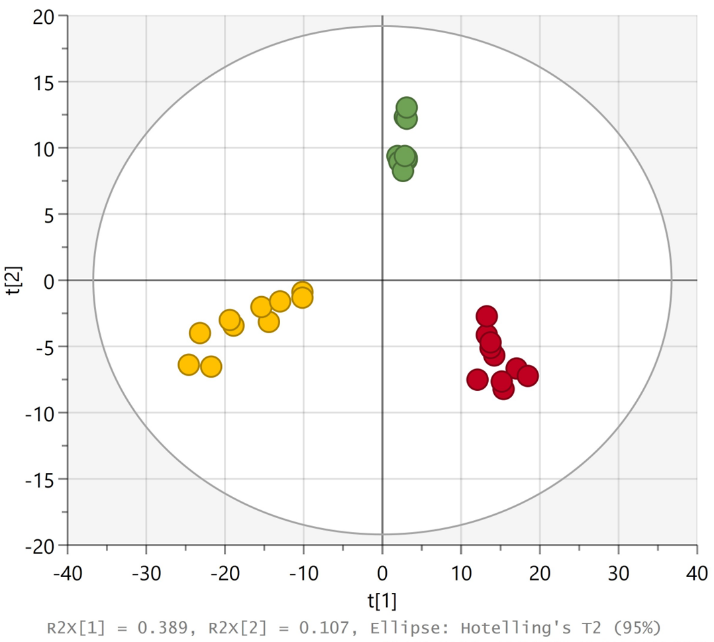


(b)

(c)


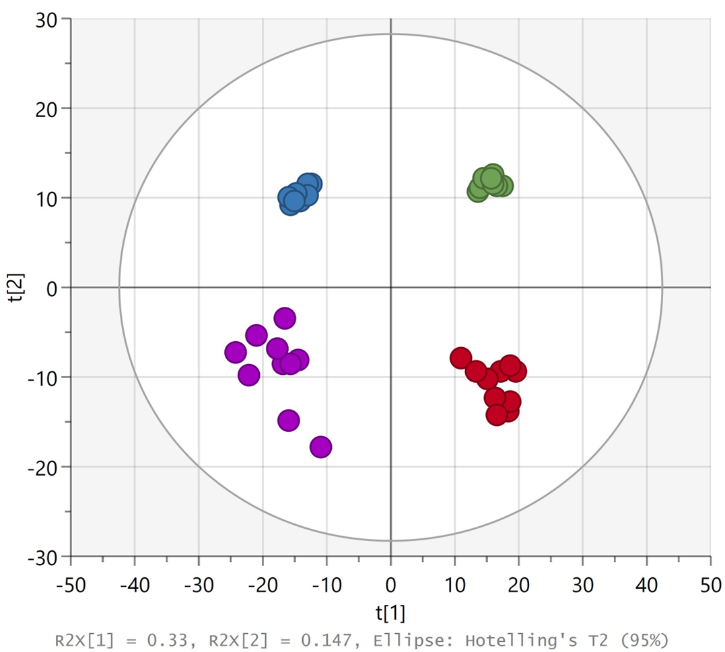

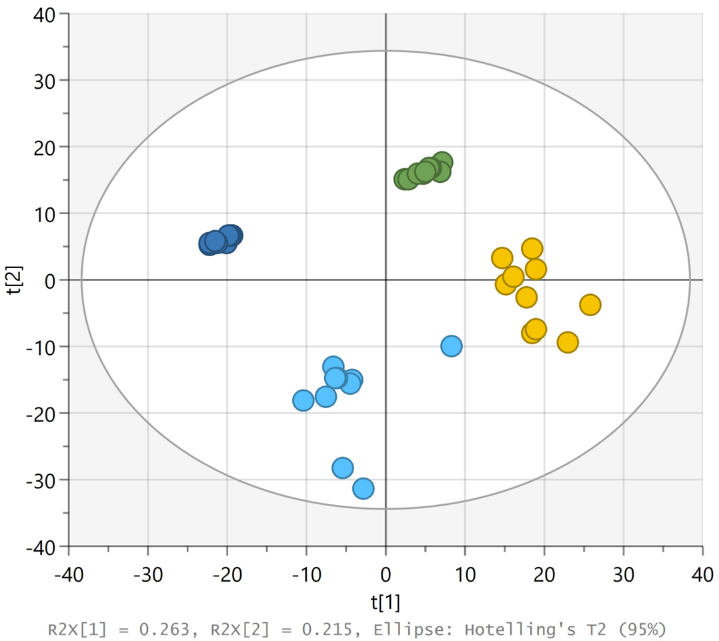


(d)

Fig S2. Metabolic pathway map comparing liver metabolomes in prespermiating (PSM) and spermiating male (SM) sea lampreys


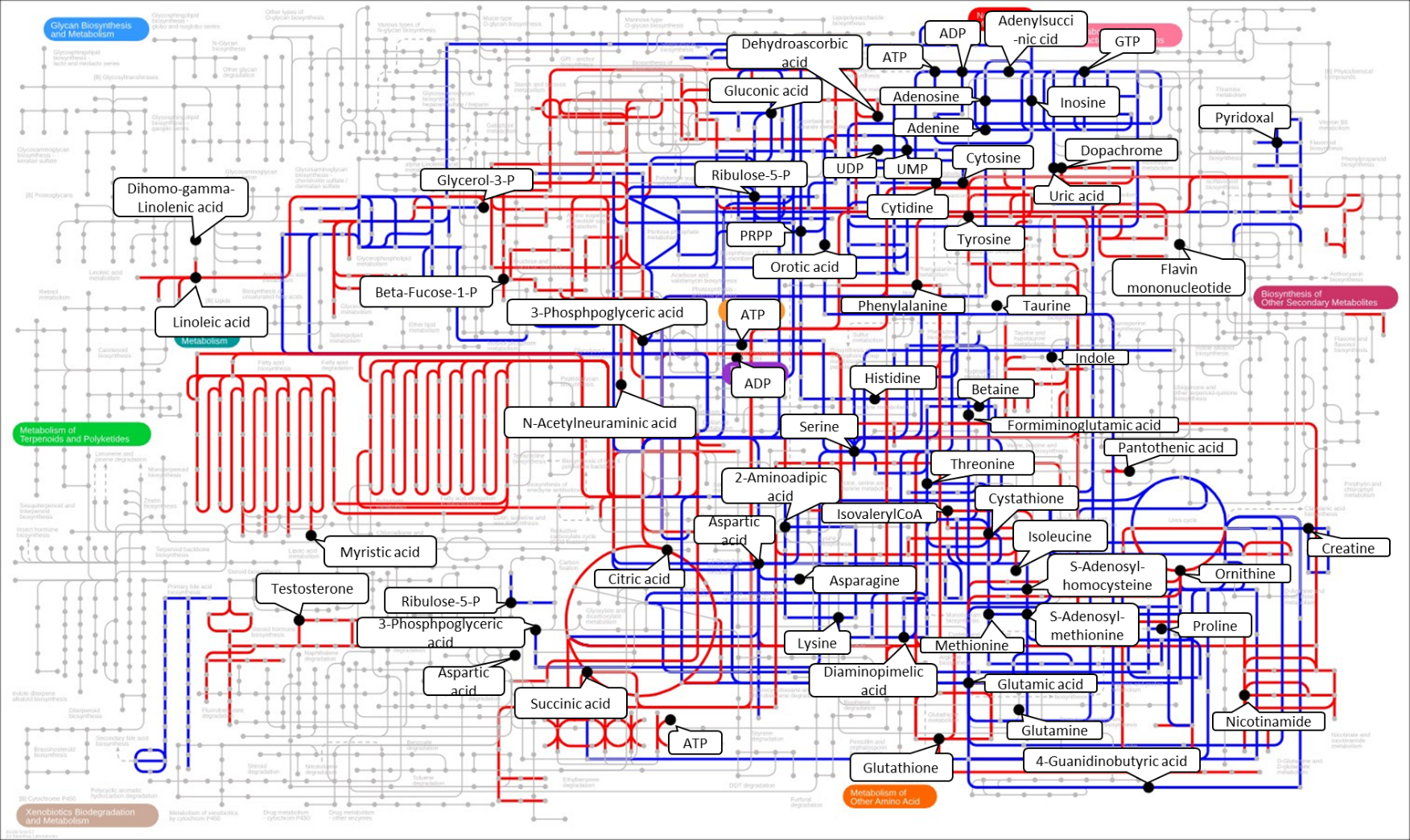


The black nodes represent the most discriminant metabolites in the group. The red and blue edges represent the upregulated (SM > PSM) and downregulated (PSM > SM) pathways, respectively. The map has been generated using Interactive Pathways Explorer (iPath) v3 (https://pathways.embl.de/).

Fig S3. Metabolic pathway map comparing liver metabolomes in preovulating (POF) and ovulating female (OF) sea lampreys


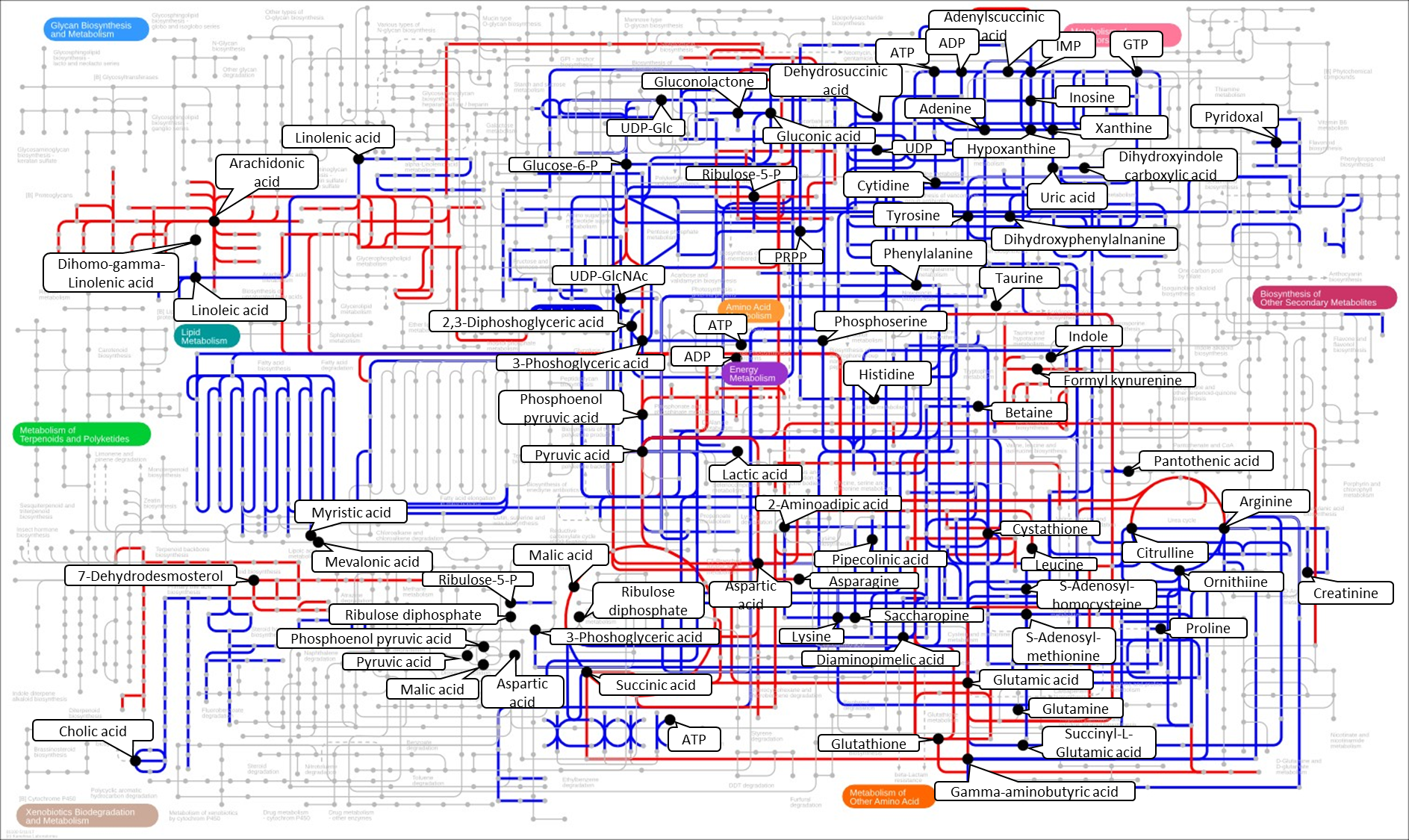


The black nodes represent the most discriminant metabolites in the group. The red and blue edges represent the upregulated (OF > POF) and downregulated (POF > OF) pathways, respectively. The map has been generated using Interactive Pathways Explorer (iPath) v3 (https://pathways.embl.de/).

Fig S4. Metabolic pathway map comparing liver metabolomes in spermiating male (SM) and ovulating female (OF) sea lampreys


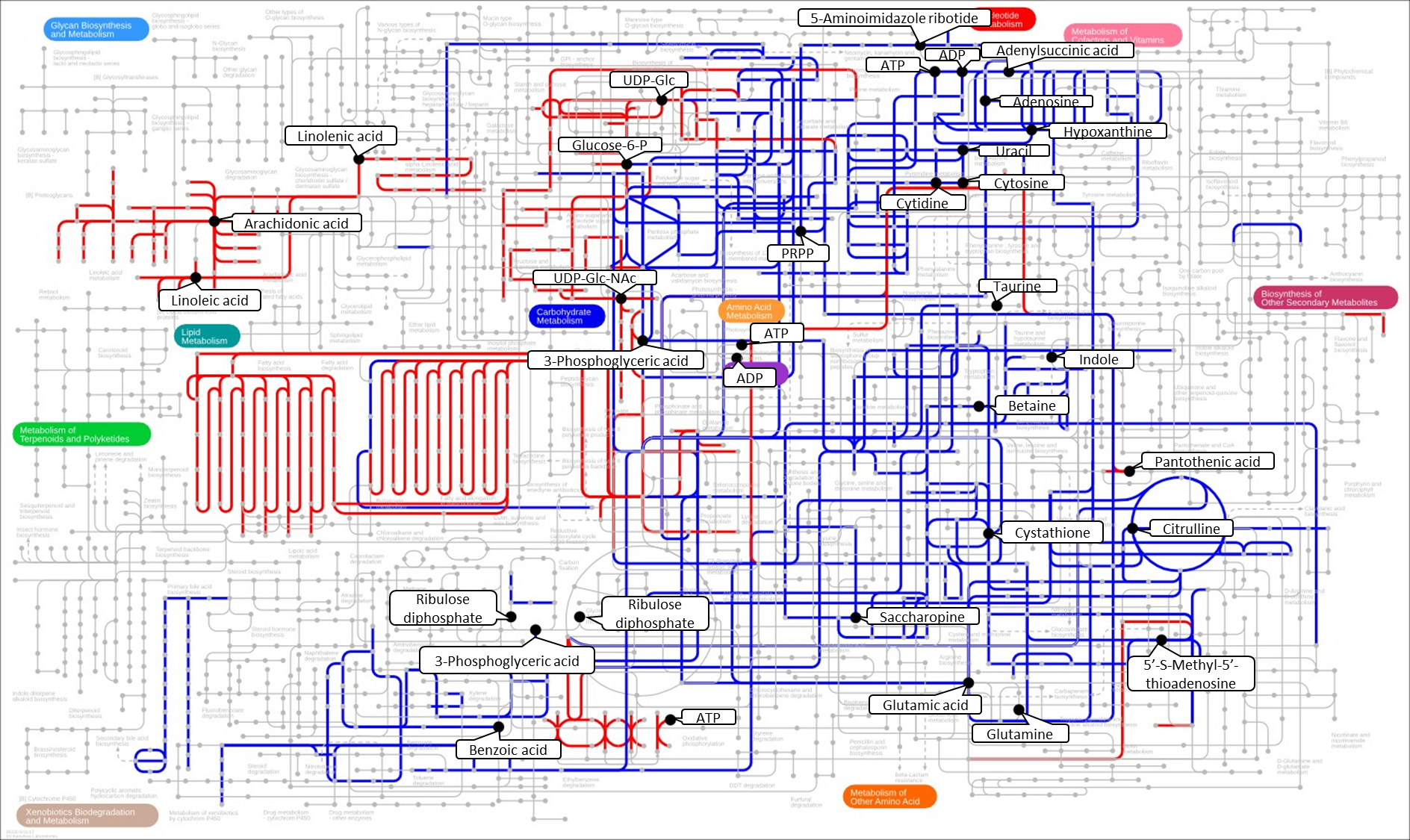


The black nodes represent the most discriminant metabolites in the group. The red and blue edges represent the upregulated (SM > OF) and downregulated (OF > SM) pathways, respectively. The map has been generated using Interactive Pathways Explorer (iPath) v3 (https://pathways.embl.de/).

Fig S5. Metabolic pathway map comparing liver metabolomes in prespermiating male (PSM) and preovulating female (POF) sea lampreys


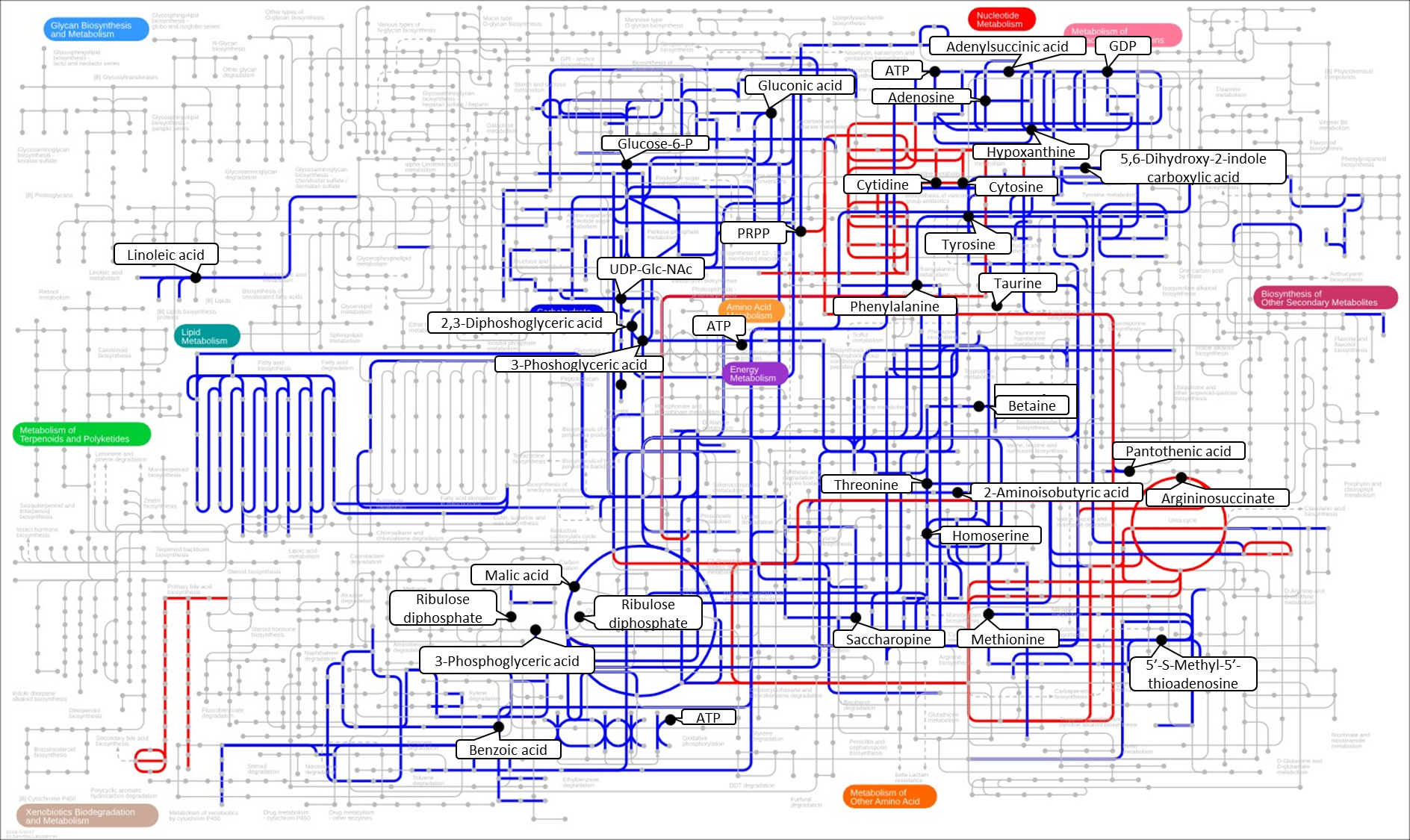


The black nodes represent the most discriminant metabolites in the group. The red and blue edges represent the upregulated (PSM > POF) and downregulated (POF > PSM) pathways, respectively. The map has been generated using Interactive Pathways Explorer (iPath) v3 (https://pathways.embl.de/).
